# Microstructures and Mechanical Properties of a Nanostructured Al-Zn-Mg-Cu-Zr-Sc Alloy under Natural Aging

**DOI:** 10.3390/ma16124346

**Published:** 2023-06-13

**Authors:** Gaoliang Shen, Zhilei Xiang, Xiaozhao Ma, Jingcun Huang, Jihao Li, Bing Wang, Zongyi Zhou, Yilan Chen, Ziyong Chen

**Affiliations:** 1 Faculty of Materials and Manufacturing, Beijing University of Technology, Beijing 100124, China; glshen@emails.bjut.edu.cn (G.S.); xiangzhilei@bjut.edu.cn (Z.X.); jchgrinm@hotmail.com (J.H.); 18810231165@163.com (J.L.); w17550209213@gmail.com (B.W.); zhouzongyi@emails.bjut.edu.cn (Z.Z.); cyl_amulet1984@hotmail.com (Y.C.); 2 Aviation Industry of China Manufacturing Technology Institute, Beijing 100015, China; maxiaozhao625@163.com

**Keywords:** high-pressure torsion, Al-Zn-Mg-Cu-Zr-Sc alloy, natural aging, microstructure, mechanical properties

## Abstract

Nanocrystalline (NC) structure can lead to the considerable strengthening of metals and alloys. Obtaining appropriate comprehensive mechanical properties is always the goal of metallic materials. Here, a nanostructured Al-Zn-Mg-Cu-Zr-Sc alloy was successfully processed by high-pressure torsion (HPT) followed by natural aging. The microstructures and mechanical properties of the naturally aged HPT alloy were analyzed. The results show that the naturally aged HPT alloy primarily consists of nanoscale grains (~98.8 nm), nano-sized precipitates (20–28 nm in size), and dislocations (1.16 × 10^15^ m^−2^), and exhibits a high tensile strength of 851 ± 6 MPa and appropriate elongation of 6.8 ± 0.2%. In addition, the multiple strengthening modes that were activated and contributed to the yield strength of the alloy were evaluated according to grain refinement strengthening, precipitation strengthening, and dislocation strengthening, and it is shown that grain refinement strengthening and precipitation strengthening are the main strengthening mechanisms. The results of this study provide an effective pathway for achieving the optimal strength–ductility match of materials and guiding the subsequent annealing treatment.

## 1. Introduction

Al-Zn-Mg-Cu alloys have been widely used for aerospace and transportation applications because of their high strength, excellent corrosion resistance, and heat treatability [1,2,3]. Among them, Al-Zn-Mg-Cu alloys micro-alloyed with Zr and Sc exhibit very good mechanical properties [4,5], and many Al_3_(Sc,Zr) particles form during solidification, which act as heterogeneous nucleation sites for Al grains, resulting in grain refinement of the alloy. Moreover, nanoscale Al_3_(Sc,Zr) particles can also precipitate homogeneously after proper heat treatment and effectively pin the grain boundaries, enhancing the strength of the Al alloy [6]. Currently, the severe plastic deformation (SPD) technique has also attracted substantial research interest for significant property enhancements since its appearance in 1991 [7,8]. To further improve the mechanical properties of the alloys, SPD processing was introduced into the Al-Zn-Mg-Cu alloys for grain refinement to produce ultrafine-grained (UFG) or nanocrystalline structures. However, NC structure alloys are facing a significant reduction in elongation according to the early plastic instability caused by their high tensile strength [9]. As such, increasing the tensile strength of the alloys while maintaining good ductility is an interesting challenge.

The high-pressure torsion process is one of the most promising SPD methods for grain refinement and can provide considerable strain to obtain uniformly nanostructured alloys. At the same time, it can refine the grain size to nanoscale, and in the process, dislocation accumulation and dynamic recovery of the alloys gradually reach a balance when the applied shear strain is very large, so the alloys will reach a stabilized state [10,11]. In recent years, two studies on HPT-processed Al 2024 [12] and Al 7075 [13] reported similar ultimate tensile strengths approaching 1 GPa. The highest tensile strength was presented in HPT-processed Al alloys, combined with elongation that generally did not exceed 2%. In addition, Mohamed et al. [14] already reported that the ultimate tensile strength of an A2024 alloy processed by HPT and subsequent aging reached 910 MPa with elongation of ~5%. Liu et al. [15] also reported that hierarchical nanostructured 2024 Al alloy processed by HPT followed by natural aging treatment resulted in an ultra-high tensile strength of 992 MPa, but the elongation was only approximately 2%. That means the HPT processing can produce very well-deformed nanostructures to obtain a desirable NC structure via annealing treatment or natural aging. However, no such investigation has yet been reported for an Al-Zn-Mg-Cu alloy with Zr and Sc additions processed by HPT with natural aging.

The purpose of this work is to clarify the microstructures and mechanical properties of the naturally aged HPT Al-Zn-Mg-Cu alloy. A nanostructured Al-Zn-Mg-Cu alloy with Zr and Sc additions was designed and successfully obtained by HPT followed by natural aging. Meanwhile, the nanostructure and underlying strengthening mechanisms of the alloy were deeply investigated, and fundamental insights into the interrelationships between grain refinement, precipitates’ characteristics, and mechanical properties are given. The results obtained in this study can offer a guide for SPD-processed alloy by subsequent annealing treatment to pursue the optimal strength–ductility match and provides excellent material selection for practical engineering applications.

## 2. Materials and Methods

**Figure 1:**
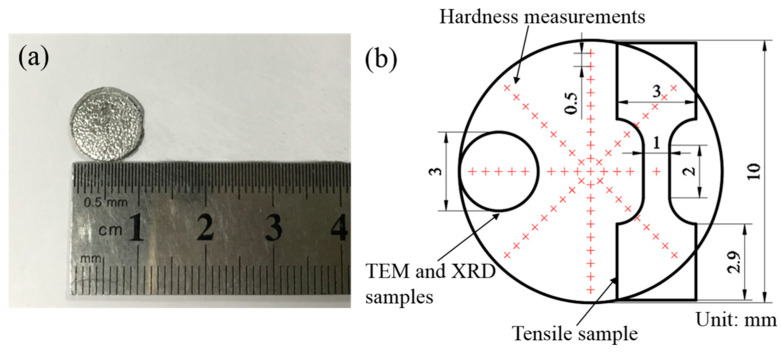
(**a**) The appearance of the disc-shaped Al-Zn-Mg-Cu-Zr-Sc alloy samples prepared by HPT. (**b**) The positions extracted for microstructures analysis, positions marked for Vickers microhardness, and the dimensions of the tensile sample.

This work was conducted on Al-Zn-Mg-Cu-Zr-Sc alloy discs with dimensions of a 10 mm diameter and 1 mm thickness. The chemical composition of the alloy is listed in Table 1. High-purity Al (99.99%), high-purity Zn (99.99%), high-purity Mg (99.9%), Al-50%Cu, Al-4%Zr, and Al-2%Sc master alloys were added as raw materials to the alloy melt at 780 °C. After all the elements had melted, hexachloroethane was used to degas and slag the alloy melt, stirring uniformly for 5 min at a casting temperature pf 730 °C, and an alloy ingot with a 90 mm diameter was prepared by permanent mold. The alloy ingot was hot extruded into a rod 20 mm in diameter with an extrusion ratio of 20.25:1 at an extrusion temperature of 410 °C. Therefore, the alloy was provided in the form of an extruded rod with a diameter of 20 mm, independently developed by our research group. After that, the alloy rod was exposed to solid-solution treatment at 465 °C for 1.5 h and then water quenched to room temperature (RT). The alloy rod was machined into the above discs and, subsequently, HPT processing was carried out at room temperature under a pressure of 6 GPa, a rotation speed of 0.5 rpm, and a rotation angle of 1800°. The appearance of the selected disc-shaped Al-Zn-Mg-Cu-Zr-Sc alloy samples prepared by HPT is shown in Figure 1a. Finally, the alloy samples prepared by HPT were then naturally aged (NA) for half a year.

In order to better analyze the results of the naturally aged HPT alloy samples, the positions extracted for microstructures analysis, positions marked for Vickers microhardness, and the shape and size of the tensile samples are depicted in Figure 1b. Based on this, we explored the microstructure and mechanical properties of the alloy in detail. The samples of the alloy were ground with abrasive paper and polished to a mirror-like surface. Subsequently, in order to characterize the phase constituents and dislocation density, X-ray diffraction (XRD, D8 ADVACEX, Bruker, Billerica, MA, USA) measurements were taken on a D8 ADVACEX diffractometer equipped with a CuKa target, with scanning angles of 10–90°and a scanning speed of 2°/min. Further details of the experimental methods of the XRD patterns are mentioned in [16,17]. The microstructures of the alloy were analyzed by a transmission electron microscope (TEM, FEI Talos F200X-G2, FEI, Portland, OR, USA) equipped with energy-dispersive spectroscopy (EDS) operated at 200 kV. At the same time, high-angle annular dark-field scanning transmission electron microscopy (STEM HADDF) images were also obtained. The alloy samples for TEM observations were prepared by Focused Ion Beam (FIB, Thermo Fisher Scientific, Waltham, MA, USA) on Helios G4 PFIB CXe, and the 3 mm diameter disks were thinned by a two-jet electropolishing technique with an electrolyte ratio of 70% methanol and 30% nitric acid at 90 mA and an applied temperature of −25 °C. The average grain size of the nanostructured alloy was determined from more than 200 grains in TEM dark-field (DF) images.

Vickers microhardness measurements were completed along 8 different radial directions of the alloy disc with an interval of 0.5 mm between two dots, and a load of 1.96 N was applied for a dwell time of 10 s using an HXD-1000TMC/LCD microhardness tester (Shanghai Taiming optical instrument Co., Ltd., Shanghai, China). The tensile tests of the alloy discs were performed at room temperature using a Shimadzu tensile testing machine (Kyoto, Japan) at a strain rate of 8.3 × 10^−4^ s^−1^, and the gauge length, width, and thickness of the dog-bone-shaped tensile samples were 2 mm, 1 mm, and 0.6 mm, respectively. Moreover, the tensile displacements and local strain distributions of the alloy disc were accurately recorded by a dual camera and data analysis by the digital image correlation (DIC) method using Vic-2D software (VIC-2D 6_x64). The tensile fracture surfaces of the alloy were observed by scanning electron microscopy (SEM, Quanta FEG 650, C528FEI, Eindhoven, The Netherlands). In order to calculate the dislocation density, the diffraction peak data of the alloy was analyzed by the modified Williamson–Hall method.

## 3. Results and Discussion

### 3.1. Phase and Microstructure

**Figure 2:**
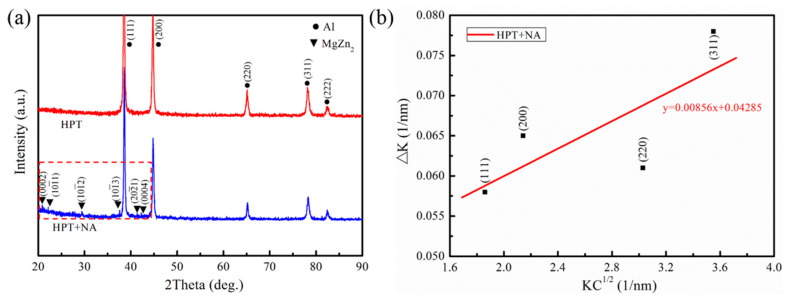
(**a**) Typical XRD patterns of the HPT and naturally aged HPT samples. (**b**) Linear fitting and the dislocation density of the naturally aged HPT sample.

The typical XRD patterns of HPT and naturally aged HPT alloy samples are shown in Figure 2a. It can be seen that the HPT alloy has only α-Al phase diffraction peaks. As for the naturally aged HPT alloy, except for weak diffraction peaks of a MgZn_2_ phase (η-phase) with a close-packed hexagonal (hcp) structure (the inset of the red framed zone in Figure 2a), other strong diffraction peaks are identified as the face-centered cubic (fcc) crystalline phase of α-Al. The lattice parameter of α-Al in the naturally aged HPT sample is 0.4041 nm, slightly below that of pure Al (0.4050 nm), which is primarily due to the Zn atoms in the α-Al lattice, and the Zn atom has a smaller atomic radius compared with the Al atom. Upon analyzing the XRD patterns, it can be concluded that many of the second phases precipitated in the alloy after natural aging for half a year. Figure 2b shows the line fitting of the XRD result of the naturally aged HPT sample. According to the modified Williamson–Hall equation in related literature [18,19], the average dislocation density of the alloy is estimated. The equation is listed as follows:

(1) $$\Delta K≅\frac{1}{D}+\sqrt{\frac{{\pi M}^{2}b^{2}\rho }{2}}{(KC}^{\frac{1}{2}}{)+o(K}^{2}C)$$

In Equation (1), D, M, and ρ are the coherent scattering domain size, dislocation arrangement parameter (M values: 1–2), and dislocation density, respectively. K is the modulus of the diffraction vector, equal to 2sinθ/λ, and θ and λ are the diffraction angle and the X-ray wavelength (λ = 0.154056 nm). ΔK equals 2Δθcosθ/λ, and Δθ is the full width at half maximum of the diffraction peak of the alloy. δ and b are the Scherrer constant (δ = 0.9) and the absolute value of Burgers vector of dislocation (b = 0.286 nm in aluminum alloy), respectively. C is the contrast factor of the dislocations (C = 0.1874), which is constrained by the elastic relative orientations among Burger’s vector, the line vector of dislocations and the diffraction vector, and the elastic constants of the materials [20]. Using the equation, the calculated dislocation density of the alloy is 1.16 × 10^15^ m^−2^, and its relationship with mechanical properties will be discussed in detail later.

**Figure 3:**
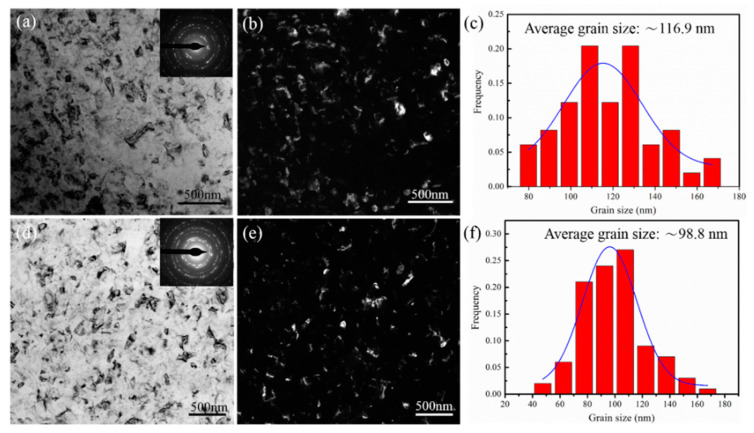
Microstructures of the HPT alloy and natural-aged HPT alloy samples. (**a**,**d**) TEM BF image; (**b**,**e**) corresponding DF image; (**c**,**f**) grain size statistics.

Figure 3a,b show the microstructures of TEM BF and DF images of the HPT alloy sample. Combined with the selected area diffraction pattern (SAED) in Figure 3a, continuous diffraction rings indicate that the Al grains of the alloy are nanoscale, and the average grain size of the HPT alloy is 116.9 ± 0.3 nm, as shown in Figure 3c. Moreover, Figure 3d,e show the microstructures of the naturally aged HPT alloy. The grain sizes of the nanostructured alloy were measured using the TEM DF images using the line interception method. Measuring over 200 grains in 30 TEM DF images, the average grain size value of the naturally aged HPT alloy is 98.8 ± 0.2 nm, and the grain size distribution is presented in Figure 3f. Compared with the HPT alloy, the grain size of the naturally aged alloy is reduced by 15.5%, indicating that recrystallization occurs in the natural aging process.

**Figure 4:**
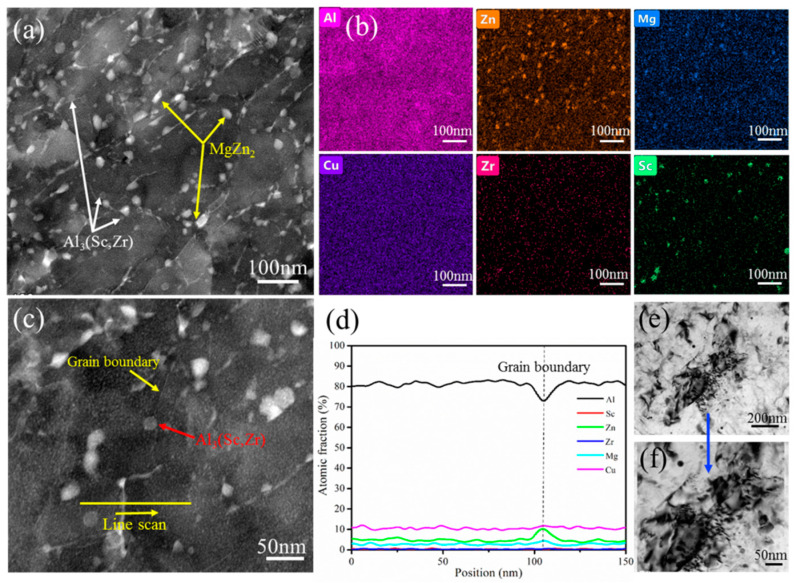
Microstructures of the naturally aged HPT alloy sample at high magnification. (**a**) STEM HADDF image. (**b**) Corresponding EDS mapping images. (**c**) STEM HADDF image. (**d**) EDS line profile obtained along the yellow arrow in (**c**). (**e**) Dislocation feature. (**f**) Enlarged area in (**e**).

The microstructures of the naturally aged HPT alloy sample at high magnification are shown in Figure 4. Figure 4a,b show the STEM HADDF micrograph and corresponding EDS elemental mapping image. It can be seen that many precipitates existed in the naturally aged HPT sample. According to the EDS elemental mapping, on one hand, the bright white precipitates should be the MgZn_2_ phase, as denoted by yellow arrows, but a small part of them appears inside the grains and most of them appear at the grain boundaries with an average size of approximately 28 nm. The volume fraction of the MgZn_2_ phase is 3.4% and the precipitation is primarily along the grain boundaries. On the other hand, the gray spherical precipitates should be the Al_3_(Sc,Zr) phase, as denoted by white arrows, but primarily appear inside the grains with an average diameter of approximately 20 nm a volume fraction of the Al_3_(Sc,Zr) phase of 1.9%. At the same time, it can be elucidated that the precipitation of the HPT alloy took place even at room temperature. As can be seen in Figure 4c,d, Figure 4c also shows the STEM HADDF image. The Al_3_(Sc,Zr) phase can pin the grain boundaries of the alloy, inhibit the movement of the grain boundary, and promote the strength of the alloy. Figure 4d shows an EDS line profile across the grain boundary in Figure 4c, confirming the enrichment of Zn and Mg solute atoms at the grain boundary. After natural aging, the solute atom enrichment of the alloy is appreciable at almost all grain boundaries, and it also shows that the precipitates at grain boundaries may be a MgZn_2_ phase. Figure 4e,f present the morphological characteristics of dislocations, Figure 4f is an enlarged image of Figure 4e (as indicated by the blue arrow), and the strength of the naturally aged HPT alloy still has the contribution of dislocation strengthening. Compared with the HPT-processed alloy sample, the dislocation density of the naturally aged HPT alloy sample is lower due to dislocation recovery and recrystallization.

**Figure 5:**
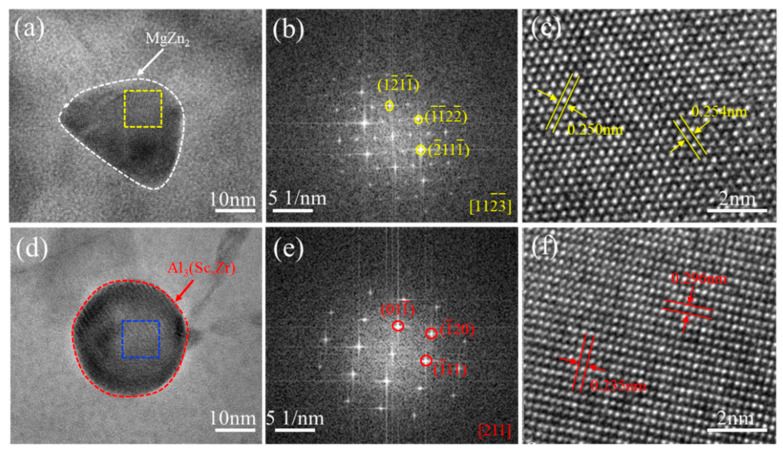
Crystal structure of the two precipitates in the naturally aged HPT alloy samples. (**a**–**c**) MgZn_2_ phase. (**d**–**f**) Al_3_(Sc,Zr) particle.

In order to further identify the precipitates accurately, Figure 5 shows the crystal structure of the two precipitates in the naturally aged HPT alloy samples. Figure 5a reveals the typical HRTEM image of the MgZn_2_ phase. It can be clearly seen that the equilibrium MgZn_2_ phase has no coherent orientation relationship with the α-Al phase [21]. The fast Fourier transformation (FFT) pattern (the yellow frame zone in Figure 5a) in Figure 5b shows $\left\{11\bar{2}\bar{3}\right\}$ reflections through the calibration of diffraction spots, indicating the precipitate of MgZn_2_. At the same time, the lattice fringes of the MgZn_2_ phase are shown in Figure 5c. The spacing between the two crystal planes of 0.254 nm and 0.250 nm corresponds to $\left(\bar{1}\bar{1}2\bar{2}\right)$ and $\left(\bar{2}11\bar{1}\right)$, respectively. In addition, Figure 5d shows the HRTEM image of the Al_3_(Sc,Zr) phase. It can be found that the Al_3_(Sc,Zr) phase also has no coherent orientation relationship with the α-Al phase, and it is different from traditional research results indicating lattice bending and disordering as a result of high-pressure torsion. The FFT image (the blue frame zone in Figure 5d) of Figure 5e shows {211} reflections, further confirming the precipitate of Al_3_(Sc,Zr). Moreover, Figure 5f reveals that the lattice fringes of Al_3_(Sc,Zr) and the spacing between the two crystal planes of 0.296 nm and 0.235 nm correspond to $\left(01\bar{1}\right)$ and $\left(\bar{1}11\right)$, respectively.

### 3.2. Hardness and Tensile Properties

**Figure 6:**
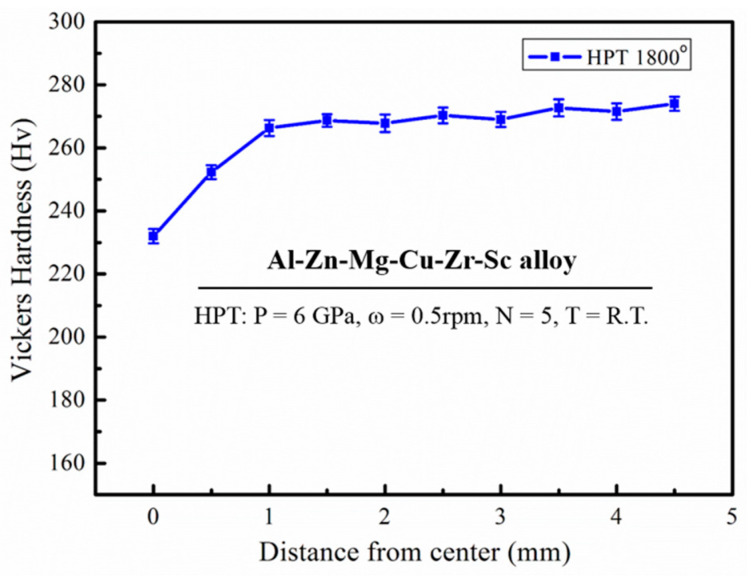
Vickers hardness against the distance from center for the HPT sample.

**Figure 7:**
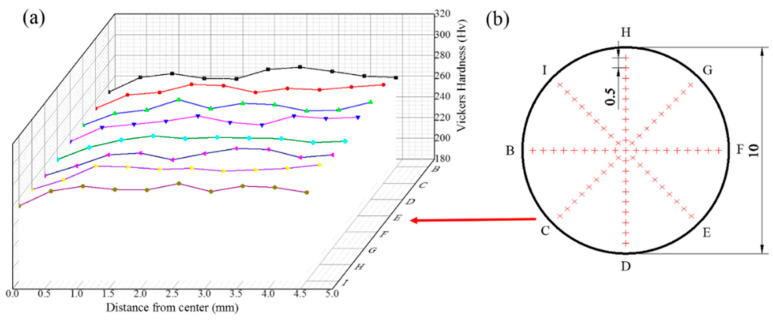
(**a**) Vickers hardness distributions of eight different radial directions and (**b**) positions marked for Vickers hardness in a naturally aged HPT disk.

Figure 6 plots the Vickers microhardness against the distance from the disc center for the HPT processed sample, it can be seen that the microhardness of the HPT processing tends to be in a stable stage of 270 HV from the disk center to the edge. Moreover, Figure 7 shows the Vickers microhardness plotted against the distance from the center along eight different radial directions at a load of 1.96 N, they are represented by the letters B-I in Figure 7b, respectively. Combined with Figure 7a,b, the hardness of the naturally aged HPT alloy sample increases with increasing distance to the center and tends toward the saturated value around 265 HV, slightly lower than the hardness of 270 HV after HPT processing (shown in Figure 6). Therefore, it also means that the microstructure of the naturally aged HPT sample is almost homogeneous. The variation of the naturally aged HPT alloy sample hardness results from the combination of nanoscale grains, precipitates, and dislocations.

**Figure 8:**
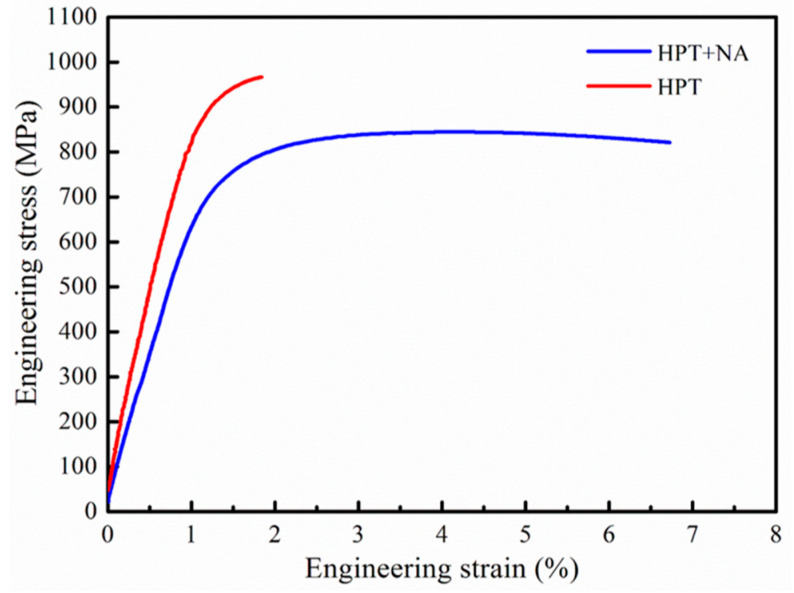
The tensile stress–strain curve of the HPT and HPT + NA alloy samples.

**Figure 9:**
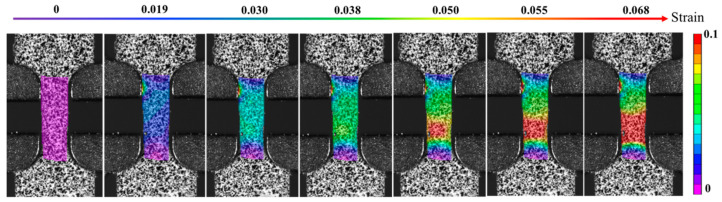
The local von Mises strain distributions of the naturally aged HPT sample at various tensile strains.

Then, the tensile mechanical properties were tested. Figure 8 shows the tensile stress–strain curves of the HPT and naturally aged HPT alloy samples at RT, and the corresponding mechanical property parameters are given in Table 2. The ultimate tensile strength (UTS), yield strength (YS), and elongation (EL) of the HPT alloy sample are 966 ± 5 MPa, 924 ± 3 MPa, and 1.8 ± 0.1%, respectively. It can be seen that the mechanical properties of the HPT-processed alloy are similar to those of previous literature [12,13,15] and belong to high strength and low elongation, while the HPT alloy is a brittle fracture. Moreover, the ultimate tensile strength, yield strength, and elongation of the naturally aged HPT alloy sample are 851 ± 6 MPa, 796 ± 5 MPa, and 6.8 ± 0.2%, respectively. Therefore, a nanostructured Al-Zn-Mg-Cu-Zr-Sc alloy with high strength and reasonable ductility is obtained by HPT followed by natural aging, and the naturally aged HPT alloy sample may be a ductile fracture, which will be discussed in the fracture characteristics section. Additionally, the local von Mises strain distributions of the naturally aged HPT sample at various tensile strains are presented in Figure 9. It was analyzed by the digital image correlation method [22] using the displacement of the observation points, and the relevant digital images before and after the deformation of the sample were calculated according to the pre-defined correlation function to obtain the tensile deformation information of the sample. At the tensile strain of 0, the localized strain value was approximately 0, presenting a uniform state. As the tensile strain increased, strain localization became more and more evident. When the tensile strain of the alloy was 0.05, the localized strain reached a high value of 0.1. Under these conditions, the neck contraction worsened, and the localized strain of the tensile sample maintained a constant of nearly 0.1 until the tensile sample fracture and the tensile strain of 0.068. In other words, the DIC technique was used to describe the process of the tensile sample.

**Figure 10:**
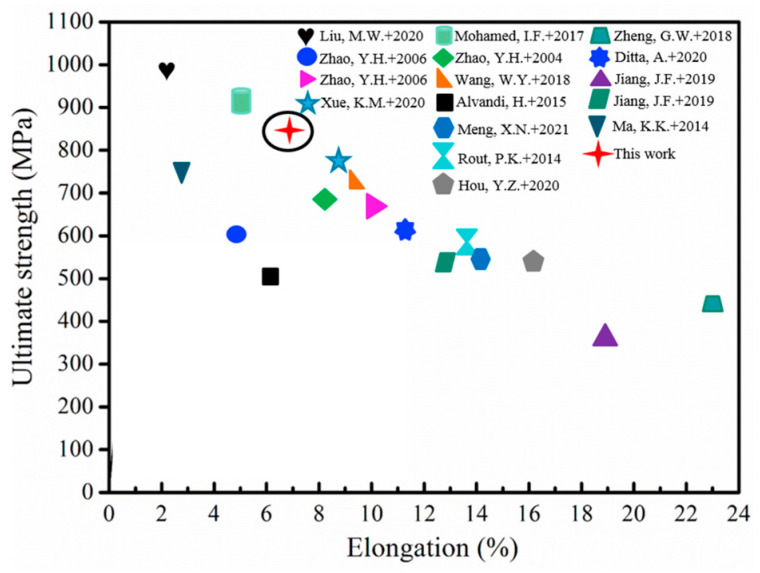
Summary of the tensile properties of high-strength Al alloys from the literature [14,15,23,24,25,26,27,28,29,30,31,32,33,34] and the present work.

The ultimate tensile strength and elongation of the naturally aged HPT sample are plotted in Figure 10, compared with the tensile properties of high-strength Al alloys from the reported literature [14,15,23,24,25,26,27,28,29,30,31,32,33,34]. It can be seen that the ultimate tensile strength of the Al-Zn-Mg-Cu alloys ranged from 450 MPa to 600 MPa under normal deformation conditions, and the elongation was relatively higher. When the other Al-Zn-Mg-Cu alloys were severely deformed followed by artificial aging treatment, the ultimate tensile strength of the alloy increased from 600 MPa to 800 MPa, while the elongation was slightly lower. Additionally, the ultimate tensile strength of a newly developed hierarchical nanostructured 2024 Al alloy processed by HPT with natural aging approached 1 GPa in the reported work, but the elongation was only approximately 2% [15]. In comparison, the ultimate tensile strength of the naturally aged HPT sample in this work is 851 ± 6 MPa and the elongation is 6.8 ± 0.2%, obtaining a reasonably high strength and ductility. The increased ductility of the naturally aged HPT alloy sample indicates that dislocation recovery of the HPT processed alloy takes place even at room temperature, and the reduction of dislocation density of the naturally aged HPT alloy provides more space for the dislocation motion and accumulation during the tensile process of the alloy and increases the elongation of the alloy [35]. According to the present work, it can provide guidance for subsequent annealing treatment in future work.

### 3.3. Fracture Characteristics

**Figure 11:**
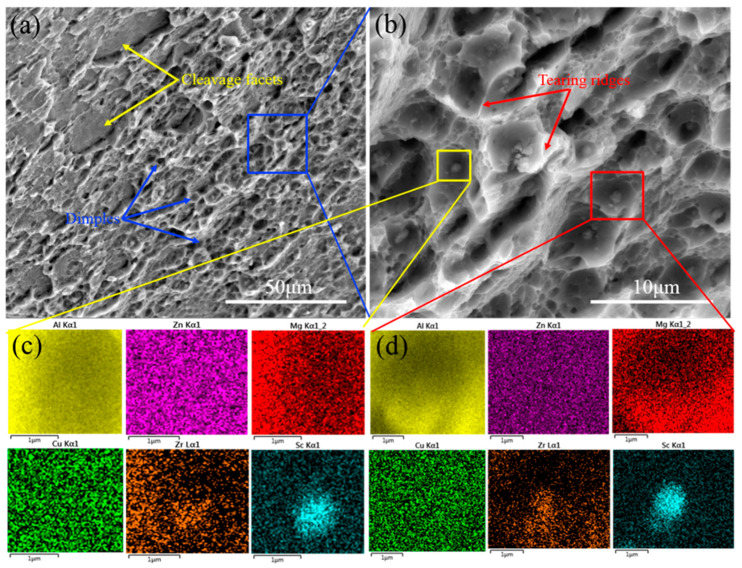
Fracture surfaces of the naturally aged HPT sample: (**a**) Low-magnification image; (**b**) high-magnification image of blue rectangle area in (**a**); (**c**,**d**) elemental mapping images of the yellow and red rectangle areas in (**b**), respectively.

In order to study the tensile fracture mechanisms of the naturally aged HPT sample, fracture surface morphologies were observed by SEM, as shown in Figure 11. As can be seen from Figure 11a, many dimples with an average size of 3 μm and cleavage facets are discovered, and the former morphology is dominant, indicating that the alloy belongs to a ductile and brittle mixed fracture, but it is primarily a ductile fracture. Figure 11b is the enlarged blue rectangle area in Figure 11a, and it can be found that the sizes of the dimples are not uneven and we observed tearing ridges around the dimples. The formation of the alloy dimples is centered on the second phase. Due to the existence of part of the second phases, these second phases may become the center of the dimples, so the size of the alloy’s dimples is not uneven in the fracture morphology of the alloys. Moreover, Figure 11c,d show the elemental mapping images of the yellow and red rectangle areas in Figure 11b, respectively. It can be elucidated that the second phases in the center of the dimples are Al_3_(Sc,Zr) particles and there may be some large phases left over from the previous solution treatment without remelting.

### 3.4. Strengthening Mechanisms

In this work, a nanostructured Al-Zn-Mg-Cu-Zr-Sc alloy under natural aging contains NCs, nano-sized precipitates, and dislocations. As a result, the multiple strengthening effects of the naturally aged HPT alloy are discussed in the following paragraphs. The contributions of grain refinement strengthening, precipitation strengthening, and dislocation strengthening to yield the strength of the alloy are calculated in order to clarify the strengthening mechanisms.

The total strength of the naturally aged HPT alloy could be estimated as [36,37,38]:

(2) $$\sigma_{YS}{=\sigma }_{i}{+\sigma }_{g}{+\sigma }_{pct}{+\sigma }_{d}$$

where $\sigma_{YS}$ is the overall yield strength of the alloy and $\sigma_{i}$ is the lattice friction stress value (35 MPa) for the Al alloy [39]. $\sigma_{g}$ is the grain refinement strengthening, $\sigma_{pct}$ is the precipitation strengthening, and $\sigma_{d}$ is the dislocation strengthening.

#### 3.4.1. Grain Refinement Strengthening

The grain refinement strengthening is generally discussed by the classical Hall–Petch formula [40,41]. The HPT processing can refine the grain size of the alloy to the nanoscale, creating a high volumetric density of grain boundaries that hinder dislocation movement and propagation, thereby strengthening the alloy [42]. It can be expressed as:

(3) $$\Delta \sigma_{g}{=\sigma }_{0}{+K}_{y}d^{-1/2}$$

where $\Delta \sigma_{g}$ is the contribution of grain refinement strengthening to the yield strength of the alloy, and $\sigma_{0}$ and $K_{y}$ are constants relating to the alloy. For the Al-Zn-Mg-Cu alloys, $\sigma_{0}$ is 16 MPa, $K_{y}$ is 0.12 MPa m^1/2^, and d is the average grain size (98.8 nm), which has been obtained by TEM as shown in Figure 3. According to the above Equation (3), with the decrease in grain sizes, the strength of the alloy increases, and the contribution value of grain refinement strengthening for the alloy is 395 MPa.

#### 3.4.2. Precipitation Strengthening

As for the naturally aged HPT alloy, the precipitates (MgZn_2_, Al_3_(Sc,Zr)) exist in the grains and grain boundaries. On one hand, the precipitates in the grains result in strength enhancement by the Orowan bypassing mechanism [16,43]. It has been determined that nanoparticles pin dislocations, resulting in dislocations bending around particles and creating Orowan rings [32]. On the other hand, combined with the related literature [44], the grain boundaries precipitates can be considered to strengthen the alloy by load transfer. Therefore, the two formulas are as follows:

(4) $$\Delta \sigma_{pct1}=M\frac{0.4Gb}{\pi \sqrt{1-\nu }}\cdot \frac{\ln(\frac{\sqrt{\frac{2}{3}}}{b}d)}{\sqrt{\frac{2}{3}}d(\sqrt{\frac{\pi }{{4V}_{p}}-1})}$$

(5) $$\Delta \sigma_{pct2}=0.5V_{p}\sigma_{YS}$$

where $\Delta \sigma_{pct1}$ and $\Delta \sigma_{pct2}$ are the contributions of precipitation strengthening to the yield strength of the alloy. In the formulas, M is the Taylor factor (3.0), G is the shear modulus (26 GPa), b is the Burgers vector (0.286 nm), ν is the Poisson ratio (0.33), d is the average size of the precipitates, and $V_{P}$ is the volume fraction of the precipitates. In addition, two kinds of precipitates existed in the naturally aged HPT alloy, and the strengthening contributions were calculated separately. Using Equations (4) and (5), the value related to precipitation strengthening for the alloy is 244 MPa.

#### 3.4.3. Dislocation Strengthening

Dislocations interact with themselves and hinder their own movement. The contribution of dislocation strengthening of the alloy can be estimated by the Bailey–Hirsch equation [45,46,47]:

(6) $$\Delta \sigma_{d}{=\alpha MGb\rho }^{1/2}$$

where $\Delta \sigma_{d}$ is the contribution of dislocation strengthening to the yield strength of the alloy, and α is 0.2. Based on the above analytical results by XRD (Figure 2), ρ is the dislocation density (1.16 × 10^15^ m^−2^). Thus, the strength contributed by dislocations is calculated to be 152 MPa.

Therefore, the contribution of each strengthening mechanism to the yield strength of the alloy is calculated based on Equations (2)–(6), and the experimental results are shown in Table 3. It can be found that the major contributions to yield strength are from grain refinement strengthening and precipitation strengthening, while dislocation strengthening also plays an important role in strengthening in this work. The calculation yield strength value (826 MPa) is slightly higher than the measured yield strength value (796 MPa). This may be because the average grain size, precipitate size, and fraction statistics have errors. On the whole, this model is relatively appropriate to predict the yield strength of the naturally aged HPT alloy.

## 4. Conclusions

In summary, a nanostructured Al-Zn-Mg-Cu-Zr-Sc alloy under natural aging conditions was obtained in this work. Microstructures and mechanical properties were investigated by means of XRD and TEM, as well as hardness tests and tensile tests. The following conclusions are drawn:

(1) The nanostructured Al alloy under natural aging contains NCs, nano-sized precipitates, and dislocations. Moreover, the naturally aged HPT alloy exhibits an ultimate tensile strength of 851 ± 6 MPa and elongation of 6.8 ± 0.2%. To the best of our knowledge, the mechanical properties of the achieved alloy showed exceptional strength-ductility performance.
(2) HPT processing can effectively refine grain size to the nanoscale. The naturally aged HPT alloy displayed dislocation recovery and recrystallization, and the average grain size was 98.8 ± 0.2 nm.
(3) The precipitation behavior of the naturally aged HPT alloy took place at room temperature. The volume fraction of the white MgZn_2_ phase with an average size of approximately 28 nm is 3.4%, and the volume fraction of the gray spherical Al_3_(Sc,Zr) phase with an average diameter of approximately 20 nm is 1.9%.
(4) The multiple strengthening mechanisms were clarified in terms of factors such as grain refinement strengthening, precipitation strengthening, and dislocation strengthening. Meanwhile, the main strengthening mechanisms of the naturally aged HPT alloy are grain refinement strengthening and precipitation strengthening, while dislocation strengthening plays an important role in strengthening.

## Figures and Tables

**Table 1:** Chemical composition of the Al-Zn-Mg-Cu-Zr-Sc alloy in this work.

Elements	Zn	Mg	Cu	Zr	Sc	Al
Wt (%)	11.0	2.0	1.2	0.12	0.2	Bal.

**Table 2:** Tensile mechanical property parameters of the HPT and HPT + NA alloy samples.

Samples State	Ultimate Strength (MPa)	Yield Strength (MPa)	Elongation (%)
HPT Natural aging	966 ± 5 851 ± 6	924 ± 3 796 ± 5	1.8 ± 0.1 6.8 ± 0.2

**Table 3:** Contributions from different strengthening mechanisms of the alloy.

Calculated Data (MPa)					Experimental Data (MPa)
$\Delta \sigma_{i}$	$\Delta \sigma_{g}$	$\Delta \sigma_{pct}$	$\Delta \sigma_{d}$	$\sigma_{YS}$	$\sigma_{YS}$
35	395	244	152	826	796

## Data Availability

The data required to reproduce these findings cannot be shared at this time as the data also form part of an ongoing study.
